# FGF21 serum levels are related to insulin resistance, metabolic changes and obesity in Mexican people living with HIV (PLWH)

**DOI:** 10.1371/journal.pone.0252144

**Published:** 2021-05-21

**Authors:** Arguiñe Ivonne Urraza-Robledo, Marta Giralt, Faviel Francisco González-Galarza, Francesc Villarroya, Alberto Alejandro Miranda Pérez, Pablo Ruiz Flores, María Elena Gutiérrez Pérez, Peré Domingo, Francisco Carlos López-Márquez

**Affiliations:** 1 Center for Biomedical Research, Medicine School Autonomous University of Coahuila (IMSS), Torreón, Mexico; 2 High Specialty Medical Unit (UMAE) # 71, Mexican Social Security Institute, Ciudad de México, Mexico; 3 Departament de Bioquímica i Biomedicina Molecular and Institut de Biomedicina (IBUB), CIBER Fisiopatología de la Obesidad y Nutrición, Universitat de Barcelona, Barcelona, Spain; 4 Center for Biomedical Research, Faculty of Medicine, Autonomous University of Coahuila, Torreón, Mexico; 5 Biomedical Research Center Medicine School Autonomous University of Coahuila, Torreón, Mexico; 6 Department of Infectious Diseases, Hospital de la Santa Creu i Sant Pau, Institut de Recerca del Hospital de la Santa Creu i Sant Pau, Barcelona, Spain; Capital Medical University, CHINA

## Abstract

**Background:**

Antiretroviral therapy has significantly improved prognosis in treatment against HIV infection, however, prolonged exposure is associated to cardiovascular diseases, lipodystrophy, type 2 diabetes, insulin resistance, metabolic alteration, as obesity which includes the accumulation of oxidative stress in adipose tissue. FGF21 is a peptide hormone that is known to regulate glucose and lipid metabolism. FGF21 is expressed and secreted primarily in the liver and adipose tissue, promoting oxidation of glucose/fatty acids and insulin sensitivity. Alterations in FGF21 may be associated with the development of insulin resistance, metabolic syndrome and cardiovascular disease. We hypothesized that FGF21 protein levels are associated with metabolic abnormalities, placing special attention to the alterations in relation to the concurrence of overweight/obesity in people living with HIV (PLWH).

**Design:**

Serum FGF21 was analyzed in 241 subjects, 160 PLWH and 81 unrelated HIV-uninfected subjects as a control group. Clinical records were consulted to obtain CD4+ cell counting and number of viral RNA copies. Serum FGF21 levels were tested for correlation with anthropometric and metabolic parameters; glucose, cholesterol, HDL, LDL, VLDL, triglycerides, insulin and indexes of atherogenesis and insulin resistance (HOMA).

**Results:**

The participants were classified into four groups: (i) PLWH with normal weight, (ii) PLWH with overweight/obesity, (iii) HIV-uninfected with normal weight, and (iv) HIV-uninfected with overweight/obesity.

Insulin levels were higher in normal-weight PLWH than in the HIV-uninfected group but not statistically significant, however, for the overweight/obesity PLWH group, insulin levels were significantly higher in comparison with the other three groups (*p*<0.0001). For FGF21, serum levels were slightly higher in the overweight/obesity groups in both patients and controls.

In HIV-infected subjects, FGF21 levels showed a strong positive correlation with triglycerides, insulin levels and insulin resistance with a p-value <0.0001. In the seronegative group, FGF21 was only correlated with weight and waist circumference, showing an important association of FGF21 levels with the degree of obesity of the individuals.

**Conclusion:**

Insulin resistance and FGF21 elevations were observed in overweight-obese PLWH. FGF21 elevation could be viewed as a compensation mechanism as, in the control group, FGF21 correlations appeared to be confined to weight and waist circumference. This can be explained based on the action of FGF21 promoting the uptake of glucose in adipose tissue. In PLWH, FGF21 was low, possibly as a result of a change in adiposity leading to a metabolic disruption.

## Introduction

Combination antiretroviral therapy (cART) has significantly improved the prognosis of human immunodeficiency virus (HIV) infection, increasing the PLWH survival rate [1]. However, prolonged exposure to cART may also cause adverse effects including mitochondrial toxicity producing an increased risk of developing cardiovascular diseases, lipodystrophy, type 2 diabetes (T2D), insulin resistance (IR), lactic acidosis, among many others diseases [1–3]. Another metabolic alteration is obesity, which implies oxidative stress due to stored fat, and risk of insulin resistance due to the accumulation of reactive oxygen species (ROS) in adipose tissue and a deregulation in the production of adipokines [4–6].

At present, there are six main types of antiretroviral inhibitors: (i) nucleoside reverse transcriptase inhibitors (NRTIs), (ii) non-nucleoside reverse transcriptase inhibitor (NNRTIs), (iii) protease inhibitors (PIs), (iv) fusion inhibitors (FIs), (v) CCR5 co-receptor inhibitors, and (vi) integrase inhibitors (IIs), also known as integrase nuclear strand transfer inhibitors or (INSTIs) [7, 8]. Nucleoside and non-nucleoside inhibitors may prevent mitochondrial DNA (mtDNA) replication and repair by interfering with enzyme DNA-polymerase-y, which is similar to the reverse transcriptase of HIV. On the other hand, the NRTIs can strongly inhibit viral DNA elongation causing irreparable damage to mtDNA during replication, formation of ROS and a decrease in ATP production, causing cellular dysfunction [9]. PIs act directly on superoxide dismutase (SOD) and nicotinamide adenine dinucleotide phosphate (NADPH) enzymes, modifying their expression, generating ROS and causing intracellular and mitochondrial damage [10]. It has been shown that PIs also promote abnormal glucose and lipid metabolism with subsequent development of metabolic syndrome [11]. For example, lopinavir and ritonavir are drugs which have been reported to present an increased risk of metabolic toxicity [12].

Fibroblast growth factor 21 (FGF21) is a peptide hormone that is known to regulate glucose and lipid metabolism. FGF21 is expressed and secreted primarily in the liver and adipose tissue, promoting oxidation of glucose and fatty acids, and insulin sensitivity [13–15]. An increase in insulin levels has been observed in obesity and type 2 diabetes [16, 17]. As such, FGF21 therapy has been studied as a treatment against cardiovascular disease, in which improvements of adipogenesis, glucose regulation, and lipid metabolism have been previously reported [18].

People living with HIV are found to present a significant increase in FGF21 levels [19]. Similarly, elevated concentrations in FGF21 are found in patients suffering from a lipodystrophy condition, which may be associated with the development of IR, metabolic syndrome and cardiovascular disease [19]. Other studies performed in animals, particularly in rats, have also found elevated FGF21 protein levels and an overexpression in T2D, IR and lipodystrophy models [20].

The aim of the present study was to investigate the association between FGF21 protein levels with metabolic abnormalities and insulin resistance in Mexican Mestizo subjects living with HIV which were under cART treatment, placing special attention to the alterations in relation to the concurrence of overweight/obesity in people living with HIV. We hypothesized that FGF21 levels are higher in PLWH, and higher among PLWH with metabolic abnormalities.

## Materials and methods

### Patients

In order to analyze the effect of obesity on FGF21 concentrations and metabolic disorders, 241 subjects, of which 160 PLWH were recruited from the Outpatient Centers for the Prevention and Care of AIDS and Sexually Transmitted Infections (CAPASITS) of Torreon, Coahuila and the Hospital Comprehensive Care Services (SAIH) of Gómez Palacio, Durango in Mexico (88 PLWH with normal weight and 72 PLWH with overweight/obesity). All subjects were treated with *cART* for at least one year. Exclusion criteria comprised patients with T2D, oncological diseases, or patients that had any kind of hormone therapy or antidiabetic agents. All participants were surveyed for sociodemographic and clinical data including age, place of residence, time of infection, and antiretroviral therapy data. Clinical records were consulted to obtain CD4+ cell counting and number of viral RNA copies. Inclusion criteria consisted of individuals 18–50 years old with a diagnosis of HIV infection, which were classified as the case group. Additionally, 81 unrelated HIV-uninfected subjects from the blood bank of these centers were included as a control group, (25 HIV-uninfected with normal weight and 56 HIV-uninfected with overweight/obesity). Inclusion criteria comprised a serologic negative test for HIV, whereas exclusion criteria included individuals with metabolic disorders such as T2D, hypertension, dyslipidemia and oncological diseases. Both groups signed an informed consent letter where they agreed to participate in the study. This study was approved by the Bioethics Committee of the Faculty of Medicine at the Autonomous University of Coahuila in Torreon, Mexico.

### Anthropometric measurements

Height, weight and waist circumference measurements of all participants were made and classified according to their body mass index (BMI) in agreement with the World Health Organization (WHO) criteria (kg/m^2^): (i) low weight (<18.5 kg/m^2^), (ii) normal weight (18.5–24.9 kg/m^2^), (iii) overweight (25–29.9 kg/m^2^) and (iv) obesity (≥ 30 kg/m^2^).

### Biochemical measurements

Blood samples were collected under fasting conditions and centrifuged at 3000 RPM for 10 minutes to obtain serum, as well as measurements for glucose (mg/dL), total cholesterol (mg/dL), high-density lipoprotein (HDL) (mg/dL), low-density lipoprotein (LDL) (mg/dL), very low-density lipoprotein (VLDL) (mg/dL), triglycerides (mg/dL) and atherogenic index. All measurements were analyzed by colorimetry in a Vitros® 250 automated dry chemistry analyzer according to the manufacturer’s characteristics.

### Homeostatic Model Assessment of Insulin Resistance (HOMA-IR)

Insulin measurement was performed using the serum sample chemoluminescence method. The accepted reference values for the insulin hormone were 2–15 μUI/mL. Subsequently, the HOMA index calculation was performed to get the IR value [21].

### Measurement of serum FGF21

The measurement of FGF21 levels was performed on the participants’ serum. Serum levels were determined in duplicate for each sample using an enzyme-linked immunoabsorbent assay (ELISA) specific for human FGF21 (Biovendor, Germany). The methodology was performed according to the manufacturer’s instructions.

### Statistical analysis

Kolmogorov-Smirnov was performed in all continuous variables to test for normality. Sociodemographic and clinical data are presented as means, standard deviations and standard error for normally distributed variables whereas for non-normally distributed data are presented as medians. T-test was used to compare mean differences. For categorical data we used chi-squared test. For group comparisons, we used ANOVA with Tukey’s post hoc test. In addition, to examine the relationship among different variables we used Pearson correlation. Also, a multiple linear regression analysis was performed for significant associations after the univariate analysis. All results were considered significant with a value p<0.05. Finally, all calculations were performed using the SPSS statistical package version 19 (IBM Corp., Armonk, NY, USA) and the GraphPad Prism 6 package (GraphPad Software, Inc., San Diego, CA).

## Results

The present study included 241 participants, 160 of whom were people with HIV/AIDS seropositive and 81 HIV/AIDS uninfected individuals. Sociodemographic population data are listed in Table 1. Both groups were paired by sex (*p* = 0.16). As shown in Table 1, the majority (90.6%) of HIV/AIDS-seropositive subjects were given a nucleoside reverse transcriptase inhibitor, followed by non-nucleoside reverse transcriptase and protease inhibitors with 56.8% and 40.6% respectively. In addition, the average viral load and average CD4+ cell for this group are also shown in Table 1. The patients with undetectable viral load represented 80.3% of studied HIV-seropositive population. According to metabolic parameters, PLWH showed higher levels of triglycerides, VLDL cholesterol, insulin and HOMA-IR than controls, and lower for total cholesterol, LDL cholesterol and FGF21. For body composition, BMI was considerably higher in controls than in PLWH (Table 1).

**Table 1:** Demographic and clinical characteristics of the study population.

	*PLWH*		*Controls*	*p-value*
	*n = 160*		*n = 81*	
*Sex*	n (%)		n (%)	
*Male*	124 (77.5)		56 (69.1)	0.16^a^
*Female*	36 (22.5)		25 (30.8)	
*Age*	39.78 ±10.78		32.45 ± 10.61	<0.0001^b^
*Duration with HIV infection (years)*	7.05 ±6.37			
*cART*				
*NRTI*	145 (90.6%)			
*NNRTI*	91 (56.8%)			
*II*	4 (2.5%)			
*PI*	65 (40.6%)			
	M±SD			
*cART (years)*	6.42±5.83			
	Median	Min	Max	
*HIV-RNA (copies mL)*	39	20	416,107	
*CD4+ cell counting (cell uL)*	452	3	1280	
*Body composition*	Mean (SE)			
*Weight*	72.13 (1.1)		81.09 (2.2)	<0.0001^b^
*Height*	1.66 (0.01)		1.69 (0.01)	0.112^b^
*BMI*	25.40 (0.4)		28.14 (0.7)	0.0006^b^
*Waist perimeter (cm)*	90.84 (1.1)		92.66 (1.8)	0.367^b^
*Metabolic parameters*				
*Glucose (mg/dL)*	89.78 (2.0)		92.10 (1.5)	0.455^b^
*Total cholesterol (mg/dL)*	174.2 (3.0)		190.3 (6.2)	0.022^b^
*Triglycerides (mg/dL)*	218.5 (16.5)		148.7 (18.3)	0.009^b^
*HDL cholesterol (mg/dL)*	45.96 (1.0)		48.96 (1.4)	0.098^b^
*VLDL cholesterol (mg/dL)*	36.85 (1.6)		26.17 (1.4)	<0.0001^b^
*LDL cholesterol (mg/dL)*	97.44 (2.4)		115.6 (5.5)	<0.0001^b^
*IA*	4.03 (0.1)		3.99 (0.1)	0.778^b^
*Insulin (Ul)*	10.70 (0.5)		5.63 (0.5)	<0.0001^b^
*HOMA-IR*	2.53 (0.1)		1.35 (0.1)	<0.0001^b^
*FGF21 (pg/mL, log)*	2.31 (0.04)		2.56 (0.02)	<0.0001^b^
*State of residence*	n (%)		n (%)	
*Coahuila*	75 (46.9)		50 (61.7)	
*Durango*	66 (41.2)		31 (38.3)	
*Others*	19 (11.9)			

NRTI: nucleoside reverse transcriptase inhibitor, NNRTI: non-nucleoside reverse transcriptase inhibitor, II: integrase inhibitor and PI: protease inhibitor.

^a^Chi-squared test and

^b^t-student test. SD: standard deviation, SE: standard error.

To analyze the biochemical parameters and their relation with the obesity, the participants were classified into four groups: (i) PLWH with normal weight, (ii) PLWH with overweight/obesity, (iii) HIV-uninfected with normal weight, and (iv) HIV-uninfected with overweight/obesity, based on BMI classification. All seropositive individuals included in the study had a regime of *cART* of at least one year, with a mean of 6.42 years under treatment (Table 1).

As shown in Table 2, most measurements demonstrated significant differences among the four groups, particularly those related to weight, BMI, waist perimeter, VLDL, LDL and HOMA-IR which showed the highest differences with a p-value<0.0001. Interestingly, insulin levels were higher in normal-weight PLWH cases than in the overweight/obesity uninfected group (7.9 vs 6.5), although not statistically significant. However, for the overweight/obesity PLWH group the differences in insulin levels were significant in comparison with the other three groups (*p*<0.0001). For FGF21, protein levels were slightly higher in the overweight/obesity groups in both patients and controls.

**Table 2:** Clinical and biochemical parameters in PLWH and HIV-uninfected individuals grouped by BMI classification.

	Controls		PLWH		
	Normal weight	Overweight/Obesity	Normal weight	Overweight/Obesity	p-value
	n = 25	n = 56	n = 88	n = 72	
**Weight**	68.0	87.1 ***aaaa***	62.7 ***bbbb***	83.5 ***aaaa cccc***	<0.0001
**Height**	1.74	1.67	1.66	1.67	0.093
**Body composition (BMI)**	22.3	30.8 ***aaaa***	21.7 ***bbbb***	29.8 ***aaaa cccc***	<0.0001
**Waist perimeter (cm)**	80.2	98.7 ***aaaa***	83.2 ***bbbb***	100.4 ***aaaa cccc***	<0.0001
**Glucose (mg/dL)**	87.1	94.4	87.1	93.0	0.393
**Total cholesterol (mg/dL)**	193.4	188.4	169.9	179.5	0.035
**Triglycerides (mg/dL)**	103	170.2	191.1	251.9 ***aa***	0.005
**HDL cholesterol (mg/dL)**	53.2	46.9	47.8	43.6	0.010
**VLDL cholesterol (mg/dL)**	20.6	28.8	32.8	41.6 ***bb aaa***	<0.0001
**LDL cholesterol (mg/dL)**	119.5	113.3	89.1 ***b***	94.1	0.0001
**IA**	3.7	4.09	3.80 ***b***	4.3	0.046
**Insulin (Ul)**	3.6	6.5	7.9	14.1 ***aaaa bbbb cccc***	0.0001
**HOMA-IR**	0.80	1.62	1.84	3.37 ***aaaa bbbb cccc***	0.0005
**FGF21 (pg/mL, log)**	2.53	2.57	2.25 ***bbbb***	2.38 ***b***	0.0003

Individuals were classified according to their body mass index (BMI): normal weight <25 BMI and overweight/ obesity ≥25 BMI; HDL: high-density lipoprotein; LD: low-density lipoprotein; VLDL: very low-density lipoprotein; IA: atherogenic index; IR: insulin resistance; FGF21, fibroblast growth factor 21. Differences between groups were analyzed by ANOVA followed by Tukey’s post hoc test. Significance: *a*: p<0.05 vs normal-weight control, *b*: p<0.05 vs obesity control, *c*: p<0.05 vs normal-weight HIV+; *aa*: p<0.01, *aaa*: p<0.001, *aaaa*: p<0.0001. FGF21 values were logarithmically transformed for statistical analysis.

**Fig 1:**
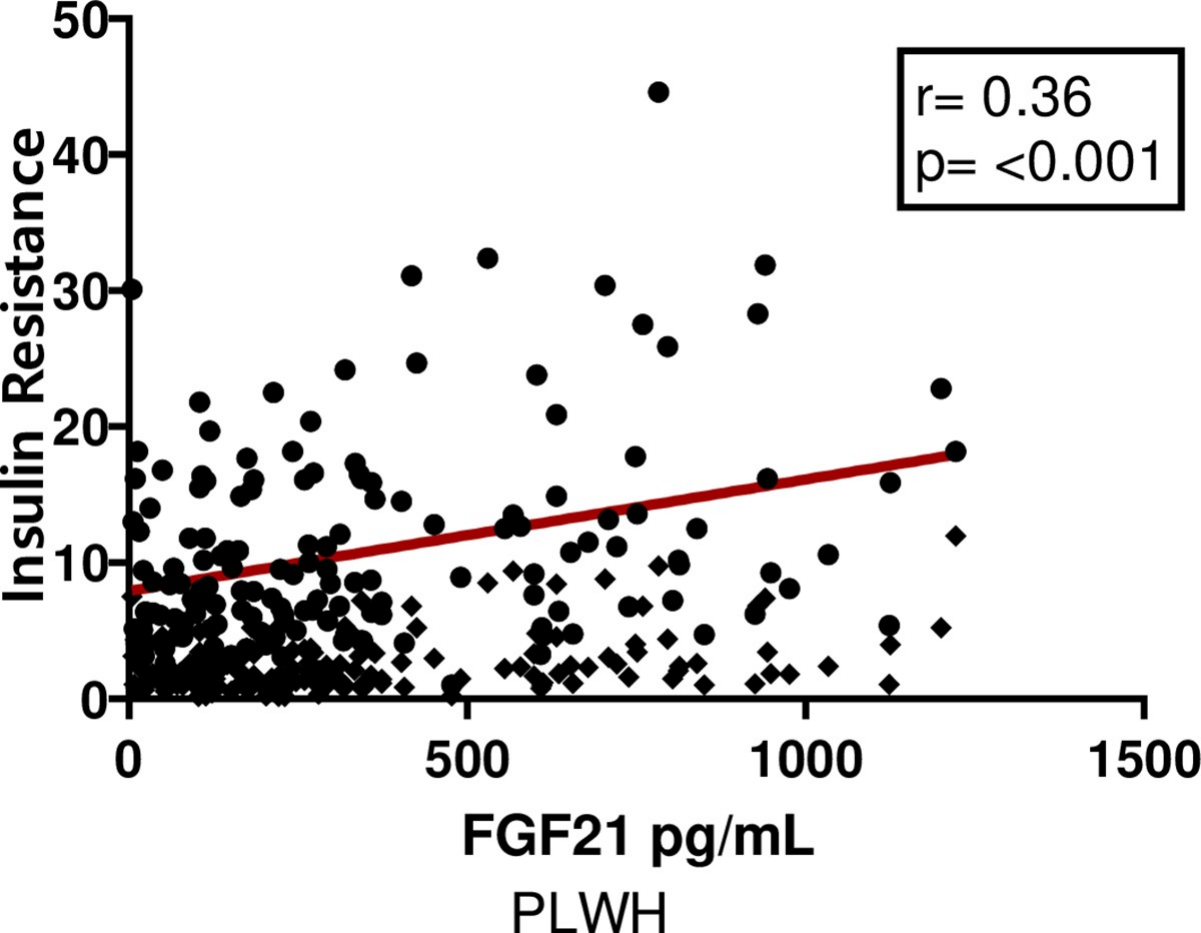
Significant correlations between insulin resistance levels and fibroblast growth factor-21 (FGF21) levels in PLWH.

In the univariate analysis, the highest correlations for PLWH subjects were observed for triglycerides, insulin levels and insulin resistance with a p-value <0.0001 for these three variables (Table 3 and Fig 1). In contrast, in the HIV-uninfected group, no correlations were found in lipid profile or in hydrocarbon metabolisms such as glucose and insulin. The only correlation was found in relation to the weight, and waist circumference, showing an important association of FGF21 protein with the degree of obesity of the individuals (Table 4).

**Table 3:** Univariate analysis for FGF21 and biochemical parameters in PLWH.

*Correlation*	*R*	*p-value*
*FGF21-Glucose*	0.23	**0.003**
*FGF21-Cholesterol*	0.09	0.253
*FGF21-Triglycerides*	0.32	**<0.0001**
*FGF21-HDL*	-0.15	0.06
*FGF21-VLDL*	0.27	**0.0005**
*FGF21-LDL*	-0.008	0.916
*FGF21-IA*	0.23	**0.002**
*FGF21-Insulin*	0.32	**<0.0001**
*FGF21-IR*	0.36	**<0.0001**
*FGF21-Weight*	0.13	0.090
*FGF21-BMI*	0.07	0.330
*FGF21-WP*	0.14	0.087

FGF21: fibroblast growth factor 21; HDL: high-density lipoprotein; LDL: low-density lipoprotein; VLDL: very low-density lipoprotein; IA: atherogenic index; IR: insulin resistance; BMI: body mass index; WP: waist perimeter. Significant values after Pearson’s correlation are shown in bold.

**Table 4:** Univariate analysis for FGF21 and biochemical parameters in HIV-uninfected individuals.

*Correlation*	*R*	*p-value*
*FGF21-Glucose*	0.11	0.326
*FGF21-Cholesterol*	0.08	0.460
*FGF21-Triglycerides*	-0.09	0.410
*FGF21-HDL*	-0.20	0.066
*FGF21-VLDL*	0.07	0.506
*FGF21-LDL*	-0.06	0.550
*FGF21-IA*	0.07	0.530
*FGF21-Insulin*	0.18	0.102
*FGF21-IR*	0.17	0.118
*FGF21-Weight*	0.26	**0.016**
*FGF21-BMI*	0.17	0.125
*FGF21-WP*	0.33	**0.003**

FGF21: fibroblast growth factor 21; HDL: high-density lipoprotein; LDL: low-density lipoprotein; VLDL: very low-density lipoprotein; IA: atherogenic index; IR: insulin resistance; BMI: body mass index; WP: waist perimeter. Significant values after Pearson’s correlation are shown in bold.

Finally, after a multivariable analysis, insulin and triglycerides showed a statistically significant correlation with serum FGF21 levels in HIV-seropositve individuals (*p* = 0.002 and *p* = 0.005, respectively) (Table 5).

**Table 5:** Multivariable analysis for FGF21 and biochemical parameters in HIV- seropositive individuals.

*Model*	*Beta*	*CI 95%*	*p-value*
*FGF21-Glucose*	0.094	(-0.695; 2.810)	0.235
*FGF21-Triglycerides*	**0.225**	(0.095; 0.538)	**0.005**
*FGF21-Insulin*	**0.244**	(3.703; 15.605)	**0.002**

FGF21: fibroblast growth factor 21; CI: confidence interval. Significant values after multiple linear regression are shown in bold.

## Discussion

The present study shows the already reported trend of worsened metabolic profile (increased glycemia, hyperinsulinemia, hypertriglyceridemia, HOMA-IR index) in PLWH, comparing these parameters with HIV-uninfected individuals stratified by BMI. Also, consistently with previous data, HIV-infected patients showed also a similar trend of metabolic abnormalities even if showing normal weight. The occurrence of overweight/obesity in PLWH worsened dramatically the metabolic profile. This is especially dramatic for the HOMA-IR index, which were highly increased in patients combining the HIV-infection and overweight/obese conditions. This indicated the high trend to insulin resistance and subsequent metabolic derangements when HIV-infected patients bear an obese condition.

Fibroblast growth factor 21, FGF21, is an endocrine factor that regulates metabolic homeostasis, and is mainly expressed in the liver. FGF21 has the potential to activate glucose uptake in adipocytes [22], being also studied in mice models to show its role in the inflammatory state of obesity [23–26]. In humans, high levels of serum FGF21 have been found in people with obesity, particularly when metabolic complications are present such as type 2 diabetes [17, 27, 28]. FGF21 high levels have also been associated with cardiovascular risk [17]. In PLWH with symptoms of lipodystrophy, serum FGF21 appears increased [13, 19, 29]. Thus, the purpose of this study was to analyze FGF21 protein levels in PLWH and to examine its relationship with metabolic changes such as insulin resistance, lipid profile disorders and inflammatory factors, paying special attention to the obesity variable.

Biochemical measurements showed significant differences between PLWH and uninfected-HIV in the lipid profile, particularly in HIV-positive groups (normal and overweight/obesity), which were similar to those found in previous studies [19, 30]. cART has been associated with dyslipidemia, which in part explains the high risk of cardiovascular complications in PLWH. Although, there are different treatments using statins for metabolic disorders such as hypercholesterolemia, these may have interactions with antiretroviral therapy in the HIV management. For example, it has been shown that some PIs and NNRTIs along with statins are metabolized by cytochrome P450 which may result in risk of intolerance [30, 31].

Insulin resistance levels in the PLWH were considerably higher compared to other HIV seronegative groups. Other studies have also reported insulin resistance in HIV-positive subjects [19, 30]. However, these previous studies did not mention the frequency or prevalence of insulin resistance. Here, we observed that 39% of the PLWH had IR.

The analysis of FGF21 showed that this protein had an increase in the control group vs. PLWH. The stratification by BMI may explain why in our results, overweight/obesity groups had higher levels of FGF21 protein, particularly in the control group; however; the number of controls used in the study may be a limitation. Therefore, it is suggested to increase the number of controls to validate these results. According to other studies, FGF21 levels were increased in a state of obesity, regardless of the degree of insulin resistance [32], although these previous studies included a patient population with a highest rate of obesity (BMI mean ~40) whereas in the current study population of overweight/obese patients averaged a BMI mean around 30. Mouse model studies and even some human studies indicated that pharmacological treatment with FGF21 can attenuate comorbidities associated with obesity [33]. Therefore, FGF21 elevation in an overweight-obese state could be viewed as a compensation mechanism or even the possibility of having resistance to FGF21.Studies performed in animal models that attempted to corroborate the signaling pathways of FGF21 and its association with insulin resistance showed that plasma elevation was due to overregulation in the liver. However, the lipodystrophic models did not allow compensation for glucose uptake and insulin resistance [20]. The present study confirmed that there is a compensation factor that may be brought by the FGF21 protein because, in the overweight/obesity control group, the insulin level remains at 6.5 UI compared to 14.1 UI observed in the overweight/obesity experimental group. FGF21 appears to act in a paracrine model to increase glucose uptake in low insulin conditions. A previous study in patients with acantosis nigricans showed an increased level of FGF21, however, the insulin levels were normal, indicating a possible compensatory response [34]. A similar trend was also seen in the present study in the control group.

It is suggested that metabolic changes may be in relation to lipid metabolism and cholesterol levels, triglycerides, especially in subjects with HIV [13]. This association was also observed in this study showing important correlations with lipid profile levels as shown in Table 3. On the other hand, there is evidence which suggests that cardiovascular disease is associated with increased levels of FGF21 [18, 35], therefore, the results found in this study showed a relationship between FGF21 and dyslipidemia in the HIV-positive population.

In the control group, FGF21 correlations appeared confined to the weight and waist circumference of the participants. This can be explained based on the action of this factor on the uptake of glucose in adipose tissue in the case of obesity or overweight [17], the elevation of FGF21 can be seen as a consequence. However, in the HIV-seropositive group, FGF21 was low, possibly as a result of a change in adiposity leading to a metabolic disruption. In addition, antiretroviral therapy has been associated for a long time with disorders such as lipodystrophy which causes a disorder in the adipose tissue distribution, mitochondrial dysfunction and alterations in adipocyte lipolysis [13].

There are multiple signaling pathways involving FGF21 such as associations with the peroxisome proliferator-activated receptors (PPARs). PPAR-γ is a transcriptional regulator of adipogenesis and inducible by FGF21 [36]. PPAR-α affects hepatic regeneration [37] and, in conjunction with the CREBH element, binds protein function as binary transcriptional activators to regulate lipid metabolism thus activating FGF21 [16]. Therefore, it is important to evaluate in the future the interaction of these signaling pathways and their relationship with metabolic changes in HIV-positive patients. In summary, circulating levels of FGF21 are associated with metabolic disturbances in PLWH, including insulin resistance, whereas in uninfected patients, FGF21 levels were mainly related to markers of visceral adiposity.

## Supporting information

S1 Data (PDF)
