# Supplementary material for: FGF21 serum levels are related to insulin resistance, metabolic changes and obesity in Mexican people living with HIV (PLWH)
Source: PLoS One. 2021 May 21;16(5):e0252144. doi: 10.1371/journal.pone.0252144 (PMC8139451; doi:10.1371/journal.pone.0252144)
Supplement: S1 Data — (PDF) [file pone.0252144.s001.pdf]

Sample	Sex	Age	Glucose	Total Choles	Triglycerid	HDL	VLDL
1	M	32	79	192	181	48	36,2
3	M	25	91	234	144	59	28,8
4	M	24	81	124	116	32	23,2
5	M	35	92	202	93	68	18,6
6	M	47	80	174	491	24	98,2
7	M	40	82	178	150	44	30
10	M	35	77	148	254	49	50,8
11	M	33	96	141	112	51	22,4
12	M	63	88	225	223	48	44,6
14	M	52	121	170	150	69	30
15	M	46	101	136	203	29	40,6
16	F	51	81	217	144	82	28,8
17	M	47	92	169	213	33	42,6
18	F	40	89	279	569	57	113,8
19	F	59	85	204	115	54	23
20	F	45	82	202	91	71	18,2
21	M	59	160	164	465	40	93
23	M	31	99	161	89	56	17,8
24	F	48	78	108	121	40	24,2
26	M	24	96	130	88	47	17,6
27	F	45	109	141	173	37	34,6
28	F	40	177	148	117	49	23,4
29	M	40	101	197	405	38	81
31	F	46	93	126	98	31	19,6
32	M	46	88	168	132	39	26,4
33	M	35	92	243	325	46	65
34	M	19	84	173	190	44	38
35	M	43	99	200	238	40	47,6
36	M	47	78	167	106	38	21,2
37	F	40	96	196	504	32	100,8
39	M	25	84	155	99	54	19,8
40	M	64	88	237	447	34	89,4
41	M	47	144	217	783	52	50
42	F	25	85	175	81	75	16,2
43	M	38	84	172	70	52	14
44	M	30	80	141	79	51	15,8
45	M	40	99	208	221	45	44,2
46	F	48	92	177	222	39	44,4
47	M	41	87	165	132	57	26,4
48	M	45	88	176	260	30	52
49	M	40	78	229	446	36	89,2
50	M	54	81	219	110	62	22
52	M	37	83	113	121	31	24,2
53	M	36	94	163	131	55	26,2
54	M	31	86	200	328	46	65,6
55	M	54	79	212	224	40	44,8

LDL	IA	Insulin (Ul)	HOMA-IR	FGF21	FGF LOG	Infection (y	cART (year
107,8	4	14,9	2,97	165,46	2,22	6	5
146,2	3,97	10,9	2,5	145,77	2,16	1	1
68,8	3,88	7,41	1,51	210,88	2,32	9	8
115,4	2,97	6,32	1,47	36,03	1,56	4	4
51,8	7,25	23,8	4,8	603,01	2,78	22	20
104	4,05	4,27	0,88	342,68	2,53	2	2
48,2	3,02	4,72	0,92	320,53	2,51	8	7
67,6	2,76	3,12	0,76	226,3	2,35	3	3
132,4	4,69	1	0,22	476,71	2,68	11	10
71	2,46	14,9	4,55	632,14	2,8	10	9
66,4	4,69	5	1,27	246,86	2,39	6	5
106,2	2,65	4,91	1	195,46	2,29	18	15
93,4	5,12	12,8	2,97	451,6	2,65	8	7
108,2	4,89	15,9	3,57	358,46	2,55	12	10
127	3,78	8,62	1,85	35,3	1,55	8	7
112,8	2,85	6,48	1,34	259,55	2,41	12	11
31	4,1	20,9	8,44	632,36	2,8	7	6
87,2	2,88	6,14	1,53	46,33	1,67	12	10
43,8	2,7	18,2	3,58	241,72	2,38	0	0
65,4	2,77	3,98	0,96	203,03	2,31	4	4
69,4	3,81	16,8	4,62	49,27	1,69	11	10
75,6	3,02	16,2	7,23	343,42	2,54	10	9
78	5,18	9,64	2,46	152,63	2,18	2	2
75,4	4,06	11,3	2,65	265,47	2,42	3	3
102,6	4,31	5,64	1,25	229,73	2,36	29	27
132	5,28	3,26	0,76	608,89	2,78	10	9
91	3,93	7,28	1,54	278,56	2,44	2	2
112,4	5	15,9	3,97	1125,12	3,05	3	2
107,8	4,39	10	1,97	266,56	2,43	2	2
63,2	6,13	28,3	6,85	929,62	2,97	8	7
81,2	2,87	2,61	0,55	53,55	1,73	3	3
113,6	6,97	8,09	1,8	976,47	2,99	31	26
115	4,17	19,7	7,16	119,87	2,08	4	3
83,8	2,33	4,56	0,98	79,42	1,9	4	2
106	3,31	6,5	1,38	271,45	2,43	7	6
74,2	2,76	4,09	0,83	10,3	1,01	3	3
118,8	4,62	30,1	7,52	4,43	0,65	11	10
93,6	4,54	31,9	7,4	940,54	2,97	7	6
81,6	2,89	24,2	5,31	319,79	2,5	2	2
94	5,87	15,5	3,44	104,65	2,02	8	7
103,8	6,36	8,73	1,72	358,23	2,55	2	2
135	3,53	3,62	0,74	177,96	2,25	7	6
57,8	3,65	8,39	1,76	60,11	1,78	25	22
81,8	2,96	1	0,24	114,08	2,06	9	8
88,4	4,35	9,51	2,06	293,12	2,47	9	8
127,2	5,3	7,12	1,42	373,57	2,57	22	20

BMI
22,41
25,8
24,34
25,1
25,51
17,11
22,86
23,45
15,79
20,55
21,11
22,21
27,04
27,64
22,32
22,22
35,88
22,38
29,64
19,96
33,15
38,75
26,03
39,45
24,3
29,19
27,46
26,93
23,92
29,4
20,83
19,66
33,46
24,44
27,78
22,86
32,41
36,21
24,86
25,36
23,89
21,49
30,82
17,27
26,15
25,18

56	M	40	73	179	218	53	43,6
57	M	34	61	227	264	33	52,8
58	M	25	84	241	204	90	40,8
59	M	30	75	276	806	33	50
60	F	39	71	192	270	53	54
61	M	28	67	140	98	50	19,6
62	M	33	95	258	722	27	50
64	M	21	71	123	171	33	34,2
65	M	40	96	184	140	43	28
66	M	38	65	152	62	71	12,4
67	F	56	77	236	236	58	47,2
68	M	41	70	102	61	38	12,2
70	F	41	74	181	91	59	18,2
71	F	29	77	156	99	51	19,8
73	M	38	86	125	300	58	60
74	M	45	97	214	327	64	65,4
75	M	38	96	235	207	52	41,4
76	M	56	73	226	81	61	16,2
77	M	51	80	153	111	50	22,2
78	M	50	81	170	195	62	39
79	M	56	78	188	252	42	50,4
80	M	54	72	166	95	59	19
81	F	56	276	218	257	42	51,4
82	M	23	91	134	102	53	20,4
83	M	40	74	148	99	43	19,8
84	M	39	82	147	101	42	20,2
85	F	31	90	125	127	35	25,4
86	M	25	74	142	174	39	34,8
88	M	45	71	146	67	52	13,4
89	M	54	83	135	118	39	23,6
90	M	51	115	189	104	50	20,8
91	F	33	89	179	110	77	22
92	M	23	73	152	98	41	19,6
93	M	29	70	129	52	45	10,4
94	M	51	261	194	1583	23	50
95	M	24	80	186	232	37	46,4
96	M	33	95	187	224	30	44,8
97	M	39	76	167	198	34	39,6
98	F	30	87	156	111	41	22,2
99	M	47	95	189	165	52	33
100	M	52	92	192	313	46	62,6
101	M	43	81	182	113	49	22,6
103	M	51	67	248	355	66	71
104	M	43	97	206	174	40	34,8
105	F	53	74	132	188	26	37,6
106	M	37	81	162	390	36	78

82,4	3,38	14,5	2,67	403,08	2,61	4	4
141,2	6,88	6,18	0,95	228,87	2,36	2	2
110,2	2,68	4,1	0,87	407,06	2,61	1	1
193	8,36	16	3,03	114,08	2,06	2	2
85	3,62	6,23	1,12	925,1	2,97	4	3
70,4	2,8	3,81	0,64	125,75	2,1	2	2
181	9,56	9,15	2,19	243,19	2,39	4	3
55,8	3,73	1	0,18	230,55	2,36	2	2
113	4,28	4,53	1,1	216,02	2,33	1	1
68,6	2,14	8,94	1,47	490,49	2,69	3	3
130,8	4,07	16,4	3,19	107,84	2,03	7	6
51,8	2,68	3,17	0,56	150,97	2,18	3	2
103,8	3,07	6,53	1,22	167,33	2,22	2	2
85,2	3,06	5,07	0,98	20,59	1,31	2	2
7	2,16	11,8	2,56	89,72	1,95	18	15
84,6	3,34	8,55	2,09	333,92	2,52	2	2
141,6	4,52	6,7	1,62	224,29	2,35	6	5
148,8	3,7	6,17	1,14	374,08	2,57	6	5
80,8	3,06	10,2	2,06	812,12	2,91	26	25
69	2,74	16,6	3,39	272,56	2,44	12	11
95,6	4,48	8,29	1,63	116,76	2,07	5	4
88	2,81	2,05	0,37	280,27	2,45	8	7
124,6	5,19	13,5	9,4	568,18	2,75	5	4
60,6	2,53	9,45	2,17	21,57	1,33	2	2
85,2	3,44	12,3	2,3	15,56	1,19	3	3
84,8	3,5	8,46	1,75	298,26	2,47	1	1
64,6	3,57	8,48	1,93	76,38	1,88	3	3
68,2	3,64	5,62	1,05	81,63	1,91	6	5
80,6	2,81	5,48	0,98	130,35	2,12	9	8
72,4	3,46	17,7	3,71	174,9	2,24	3	3
118,2	3,78	6,44	1,87	635,36	2,8	2	2
80	2,32	6,34	1,42	358,86	2,55	9	8
91,4	3,71	3,53	0,65	16,91	1,23	3	3
73,6	2,87	6,28	1,11	98,91	2	3	3
121	8,43	18,2	11,98	1221,9	3,09	5	4
102,6	5,03	3,68	0,74	174,04	2,24	1	1
112,2	6,23	6,16	1,48	26,7	1,43	1	1
93,4	4,91	9,51	1,82	223,73	2,35	11	10
92,8	3,8	11,2	2,46	292,26	2,47	1	1
104	3,63	7,15	1,71	95,94	1,98	2	2
83,4	4,17	13,2	3,06	708,74	2,85	9	8
110,4	3,71	9,61	1,96	66,1	1,82	12	11
111	3,76	4,25	0,72	345,38	2,54	8	7
131,2	5,15	7,94	1,94	166,33	2,22	1	1
68,4	5,08	3,28	0,61	121,79	2,09	1	1
48	4,5	18,2	3,72	12,99	1,11	10	9

23,53
23,94
21,77
31,4
22,52
19,27
26,37
20,81
24,45
20,8
30,29
21,3
35,9
20,83
23,83
23,04
26,78
23,8
17,96
36,2
23,38
23,03
33,33
24,07
18,93
22,16
26,02
25,34
30,8
28,54
21,72
29,72
20,91
25,61
20,42
18,15
19,43
23,66
28,4
22,32
27,61
28,4
23,36
22,1
22,29
33,76

110	M	38	65	168	95	60	19
111	M	49	103	273	1455	34	50
112	M	46	75	189	176	39	35,2
118	M	34	74	155	74	48	14,8
119	F	20	72	190	266	42	53,2
120	F	33	78	151	69	51	13,8
121	M	41	75	205	103	41	20,6
122	M	30	89	161	65	48	13
124	F	31	82	136	212	40	42,4
126	M	43	96	220	127	49	25,4
127	M	54	88	150	183	29	36,6
128	M	42	101	169	89	49	17,8
129	F		90	172	55	69	11
130	M	56	87	168	179	52	35,8
131	M	44	88	129	195	35	39
132	M	27	73	108	80	33	16
134	F	39	115	160	96	56	19,2
136	M	24	107	163	120	44	24
137	F	42	87	168	143	50	28,6
139	F	25	88	161	21	33	4,2
140	M	48	94	172	486	43	97,2
141	M	24	115	146	78	77	15,6
143	M	40	135	73	194	30	38,8
145	M	38	84	120	200	30	40
146	M	25	82	131	294	38	58,8
147	M	43	91	124	109	31	21,8
148	M	52	96	184	121	55	24,2
150	M	53	100	214	435	36	87
151	M	34	94	157	185	39	37
155	M	36	100	202	100	43	20
156	M	27	84	195	86	50	17,2
157	M	48	82	203	412	29	82,4
158	M	45	84	178	62	57	12,4
162	F	20	82	143	117	31	23,4
164	M	31	77	145	136	54	27,2
165	F	27	74	140	329	20	65,8
166	M	40	76	184	151	63	30,2
167	M	53	72	205	234	69	46,8
168	M	43	90	156	184	26	36,8
169	M	30	91	172	403	33	80,6
170	F	55	84	175	139	52	27,8
171	M	45	86	156	157	38	31,4
172	M	44	73	219	113	56	22,6
173	M	45	82	186	141	66	28,2
174	M	54	90	237	241	57	48,2
175	M	61	67	204	182	53	36,4

89	2,8	2,69	0,44	122,64	2,09	1	1
189	8,03	17,3	4,5	334,6	2,52	4	4
114,8	4,85	5,72	1,08	294,15	2,47	8	7
92,2	3,23	7,32	1,37	93,39	1,97	3	3
94,8	4,52	20,4	3,71	269,13	2,43	1	1
86,2	2,96	5,13	1,01	120,93	2,08	1	1
143,4	5	4,27	0,81	316,82	2,5	8	7
100	3,35	6,02	1,35	181,75	2,26	1	1
53,6	3,4	6,81	1,41	311,11	2,49	7	6
145,6	4,49	9,86	2,39	813,35	2,91	3	3
84,4	5,17	4,59	1,02	71,24	1,85	19	18
102,2	3,45	4,66	1,19	611,85	2,79	8	7
92	2,49	3,68	0,84	53,25	1,73	15	14
80,2	3,23	44,6	9,79	782,9	2,89	16	15
55	3,69	7,68	1,7	105,51	2,02	3	3
59	3,27	1	0,18	102,94	2,01	1	1
84,8	2,86	14	4,06	30,98	1,49	6	5
95	3,7	16,2	4,37	9,57	0,98	4	4
89,4	3,36	7,88	1,73	184,32	2,27	5	5
123,8	4,88	5,69	1,26	84,09	1,92	1	1
31,8	4	4,79	1,14	656,63	2,82	9	8
53,4	1,9	30,4	8,82	704,09	2,85	7	6
4,2	2,43	15,4	5,24	181,46	2,26	6	5
50	4	3,13	0,66	23,27	1,37	4	3
34,2	3,45	5,91	1,22	67,82	1,83	1	1
71,2	4	1	0,23	203,17	2,31	4	4
104,8	3,35	13	3,15	6,14	0,79	12	11
91	5,94	10,2	2,57	110,65	2,04	26	25
81	4,03	6,77	1,61	739,02	2,87	3	3
139	4,7	13,6	3,43	751,56	2,88	6	5
127,8	3,9	10,5	2,23	136,35	2,13	3	3
91,6	7	12,1	2,5	311,97	2,49	10	9
108,6	3,12	6,93	1,47	127,78	2,11	1	1
88,6	4,61	16,1	3,33	184,32	2,27	1	1
63,8	2,69	1	0,19	221,16	2,34	2	2
54,2	7	12,7	2,37	578,39	2,76	10	10
90,8	2,92	16,5	3,16	340,24	2,53	1	1
89,2	2,97	10,9	1,98	162,05	2,21	16	15
93,2	6	16,1	3,66	259,71	2,41	12	11
58,4	5,21	11,8	2,71	113,22	2,05	10	10
95,2	3,37	24,7	5,23	425,05	2,63	3	3
86,6	4,11	22,5	4,88	213,45	2,33	5	5
140,4	3,91	7,31	1,35	112,36	2,05	10	10
91,8	2,82	7,98	1,65	106,63	2,03	17	16
131,8	4,16	21,8	4,95	104,65	2,02	17	16
114,6	3,85	25,9	4,38	796,34	2,9	16	15

20,01
29,41
27,34
24,64
34,96
19,04
26,82
19,33
26,62
23,1
23,45
25,26
22,94
36,64
25,55
23,46
23,53
27,28
31,09
19,48
24,8
20,28
29,54
20,62
24,42
21,53
29,44
22,15
27,34
31
21,91
25,03
18,48
19,81
24,8
21,02
21,5
21,38
27,44
24,34
34,21
33,19
31,38
22,15
30,8
28,69

176	M	26	82	197	193	69	38,6
177	M	29	87	172	251	36	50,2
181	F	47	81	206	101	62	20,2
203	F	47	91	183	165	50	33
208	M	41	90	209	397	41	50
209	M	32	86	158	85	49	17
216	F	31	71	207	229	70	45,8
217	M	21	72	48	75	13	15
218	M	22	172	140	104	51	21
219	M	56	81	182	533	32	50
223	M	49	82	190	366	31	50
224	M	48	84	179	303	43	50
227	F	32	91	200	988	30	50
228	M	24	74	157	176	35	35,2
229	M	70	104	123	110	37	22
230	M	24	79	120	218	32	43,6
232	M	40	80	224	133	50	26,6
233	F	33	90	163	140	43	28
237	M	27	91	178	154	52	30,8
238	M	38	98	88	150	23	30
239	M	39	80	161	292	42	50
240	F	44	89	132	179	53	35,8

89,4	2,86	5,16	1,07	7,85	0,89	1	1
85,8	4,78	31,1	6,83	418,19	2,62	2	2
123,8	3,32	6,4	1,31	24,98	1,4	12	11
100	3,66	14,7	3,37	363,77	2,56	17	16
118	5,1	17,8	4,04	748,87	2,87	4	4
92	3,22	4,74	1,03	850,07	2,93	1	1
91,2	2,96	12,5	2,24	555,43	2,74	1	1
20	3,69	9,19	1,67	598,41	2,78	1	1
68	2,75	7,64	3,32	598,41	2,78	1	1
100	5,69	7,24	1,48	803,5	2,9	1	1
109	6,13	12,5	2,59	839,33	2,92	22	21
86	4,16	16,2	3,43	944,11	2,98	13	12
120	6,67	22,8	5,23	1200,25	3,08	11	10
87	4,49	5,24	0,98	610,06	2,79	2	1
64	3,32	32,4	8,5	530,35	2,72	7	7
44,4	3,75	5,4	1,08	1124,12	3,05	2	2
147,4	4,48	11,5	2,32	679,02	2,83	16	15
92	3,79	10,75	2,44	653,04	2,81	1	1
95,2	3,42	11,2	2,57	721,11	2,86	1	1
35	3,83	27,5	6,8	759,62	2,88	22	21
69	3,83	9,27	1,87	948,59	2,98	8	8
43,2	2,49	10,6	2,38	1033,67	3,01	7	7

20,77
38,89
24,45
35,46
34,95
24,41
32,13
24,93
24,46
23,53
32,65
25,83
24,98
17,69
23,81
28,4
24,32
23,15
26,82
31,46
28,39
25,6

Sample	Sex	Age	Glucose	Total Choles	Triglycerid	HDL	VLDL
1	M	21	87	154	76	39	15,2
2	M	25	82	135	105	32	21
3	M	29	83	265	222	58	44,4
4	M	30	85	185	125	43	25
5	M	28	80	130	140	31	28
6	M	36	94	131	84	34	16,8
7	M	32	76	114	70	36	14
8	M	18	71	177	117	57	23,4
10	M	42	143	129	223	35	44,6
11	M	28	88	162	94	45	18,8
15	M	48	86	158	159	48	31,8
17	M	31	97	217	335	43	67
18	M	41	107	141	98	38	19,6
19	M	27	83	165	227	41	45,4
20	M	20	90	140	137	41	27,4
21	M	45	87	156	131	42	26,2
23	M	28	82	147	92	43	18,4
24	M	26	99	197	254	44	50
25	M	27	91	131	87	44	17,4
26	M	31	88	158	60	45	12
27	M	24	82	181	164	49	32,8
28	M	18	91	153	115	40	23
29	M	47	88	179	382	29	50
30	M	45	95	239	162	42	32,4
32	M	27	100	161	123	40	24,6
33	M	43	97	196	131	39	26,2
35	M	28	98	147	107	40	21,4
38	M	47	88	185	325	46	65
39	M	41	93	122	98	44	19,6
40	M	42	104	233	198	52	39,6
41	M	49	97	152	87	39	17,4
44	F	43	85	192	127	63	25,4
46	F	30	81	193	98	45	19,6
47	F	28	91	145	168	37	33,6
48	F	51	90	180	110	42	22
49	F	55	108	300	187	71	37,4
50	F	44	95	254	1479	43	50
51	F	41	92	208	327	44	65,4
52	F	41	165	208	327	44	50
53	M	30	77	138	48	34	9,6
54	F	28	81	199	136	45	27,2
55	M	27	83	184	206	39	41,2
56	M	37	93	217	258	43	51,6
57	F	0	78	227	99	67	19,8
58	M	28	86	173	88	59	17,6
59	M	0	79	194	155	55	31

LDL	IA	Insulin (Ul)	HOMA-IR	FGF21	BMI
99,8	3,95	14,7	3,23	1013,07	24,11
82	4,22	10	2,07	542,89	25,13
162,6	4,57	11,1	2,32	909,18	30,12
117	4,3	8	1,72	452,43	31,1
71	4,19	3,55	0,72	437,21	23,62
80,2	3,85	10,3	2,44	475,72	24,8
64	3,17	5,23	1	530,35	26,03
96,6	3,11	3,43	0,61	452,43	20,34
49,4	3,69	14	5,05	1194,87	32,87
98,2	3,6	8,45	1,88	940,53	43,77
78,2	3,29	2	0,43	403,18	22,53
107	5,05	2,62	0,64	585,88	33,66
83,4	3,71	3,66	0,99	634,24	25,39
78,6	4,02	2,41	0,5	544,68	26,73
71,6	3,41	4,57	1,04	497,21	33,97
87,8	3,71	5,64	1,24	567,96	30,85
85,6	3,42	2	0,41	656,63	24,34
103	4,48	4,06	1,01	805,29	26,81
69,6	2,98	2,28	0,52	913,66	19,45
101	3,51	2	0,44	456,02	22,49
99,2	3,69	3,71	0,77	569,76	26,4
90	3,83	3,66	0,84	551,84	26,23
100	6,17	5,11	1,13	283,17	31,77
164,6	5,69	9,26	2,22	219,58	28,04
96,4	4,03	5,86	1,48	429,15	30,86
130,8	5,03	3,5	0,86	249,14	26,03
85,6	3,68	3,75	0,93	419,3	30,12
74	4,02	7	1,55	612,74	27,77
58,4	2,77	16,2	3,8	449,75	29
141,4	4,48	8,6	2,26	537,51	36,13
95,6	3,9	9,98	2,44	546,47	38,3
103,6	3,05	19,2	4,12	596,62	34,55
128,4	4,29	2	0,41	293,02	22,59
74,4	3,92	2,58	0,59	97,78	25,12
116	4,29	2	0,45	458,1	31,59
191,6	4,23	2	0,54	128,7	26,56
191,6	5,91	17,6	4,22	149,5	25,7
98,6	4,73	11,2	2,6	179,2	26,31
114	4,73	18,7	7,78	388,2	27,89
94,4	4,06	2	0,39	386,8	28,28
126,8	4,42	2	0,41	155,9	21,82
103,8	4,72	6,39	1,34	154	32,37
122,4	5,05	8,83	2,07	547,1	27,66
140,2	3,39	2,4	0,47	211,8	26,01
96,4	2,93	2	0,43	105,2	28,37
108	3,53	10,6	2,11	753,4	26,84

88	F	19	85	246	93	69	18,6
89	M	22	91	218	93	94	18,6
90	M	21	82	248	93	70	18,6
91	F	21	89	160	93	62	18,6
92	M	22	87		93	55	18,6
93	M	21	91	187	93	50	18,6
94	F	30	103	197	93	65	18,6
95	M	34	79	267	93	65	18,6
96	M	22	78	135	93	57	18,6
97	F	20	96	205	93	55	18,6
98	M	20	104	343	93	81	18,6
99	M	20	95	284	93	60	18,6
100	M	19	93	174	93	44	18,6
101	M	24	76	246	93	60	18,6
102	F	21	83	200	93	81	18,6
104	M	21	87	177	93	48	18,6
107	M	21	85	140	93	54	18,6
108	F	20	86	173	93	49	18,6
109	F	50	108	236	93	84	18,6
110	M	26	77	175	93	68	18,6
111	F	50	96	416	93	61	18,6
112	F	35	92	279	93	57	18,6
114	F	34	92	230	93	42	18,6
115	F	25	96	183	93	43	18,6
116	M	24	93	108	93	28	18,6
117	F	44	111	349	93	51	18,6
118	M	20	85	157	93	46	18,6
119	M	42	120	144	93	31	18,6
120	M	38	99	226	93	44	18,6
121	M	49	97	204	126	54	25,2
122	M	27	97	129	91	38	18,2
123	F	43	92	139	158	36	31,6
124	F	48	108	178	115	55	23
125	M	33	94	188	202	29	40,4
126	F	31	97	134	111	50	22,2

158,4	3,57	2	0,43	171,22	19,17
105,4	2,32	2	0,46	277,8	20,56
159,4	3,54	3	0,62	365,56	22,86
79,4	2,58	4	0,9	308,25	22,28
	0	2	0,44	284,06	21,36
118,4	3,74	2	0,46	388,85	24,36
113,4	3,03	4	1,04	512,44	27,02
183,4	4,11	2	0,4	294,81	20,26
59,4	2,37	4	0,79	397,8	24,03
131,4	3,73	4	0,97	630,66	21,66
243,4	4,23	3	0,79	306,45	22,04
205,4	4,73	2	0,48	241,08	23,67
111,4	3,95	3	0,7	341,38	19,08
167,4	4,1	3,9	0,75	462,29	22,58
100,4	2,47	2	0,42	156	26,51
110,4	3,69	3	0,66	337,8	25
67,4	2,59	3,5	0,75	334,22	26,99
105,4	3,53	9,9	2,15	283,17	30,32
133,4	2,81	2	0,54	276	29,03
88,4	2,57	5,8	1,13	417,51	39,07
336,4	6,82	5,6	1,36	402,28	31,37
203,4	4,89	3,28	0,76	454,23	27,9
169,4	5,48	2	0,46	570,65	36,07
121,4	4,26	2	0,48	570,65	44,06
61,4	3,86	9,94	2,33	344,07	50,08
279,4	6,84	2	0,56	825,89	28,52
92,4	3,41	2	0,43	348,55	33,55
94,4	4,65	2	0,61	318,99	54,09
163,4	5,14	2	0,5	734,54	28,29
124,8	3,78	2,19	0,54	94,2	22,32
72,8	3,39	4,47	1,09	93,31	25,77
71,4	3,86	9,58	2,22	174,8	36,14
100	3,24	18,9	5,15	268,5	30,84
118,6	6,48	8,45	2	108,8	24,54
61,8	2,68	8,93	2,19	56,6	26,94
